# Can transcutaneous electrical nerve stimulation improve achilles tendon healing in rats?

**DOI:** 10.1590/bjpt-rbf.2014.0107

**Published:** 2015-09-01

**Authors:** Roberta A. C. Folha, Carlos E. Pinfildi, Richard E. Liebano, Érika P. Rampazo, Raphael N. Pereira, Lydia M. Ferreira

**Affiliations:** 1 Departamento de Cirurgia Plástica, Universidade Federal de São Paulo (UNIFESP), São Paulo, SP, Brazil; 2 Departamento de Ciências do Movimento Humano, UNIFESP, Santos, SP, Brazil; 3 Departamento de Fisioterapia, Universidade Cidade de São Paulo (UNICID), São Paulo, SP, Brazil; 4 Programa de Pós-graduação em Ciências do Movimento Humano, Universidade Metodista de Piracicaba (UNIMEP), Piracicaba, SP, Brazil

**Keywords:** tendon injuries, tendinopathy, TENS, physical therapy modalities, physical agents

## Abstract

**BACKGROUND:**

Tendon injury is one of the most frequent injuries in sports activities. TENS is a physical agent used in the treatment of pain but its influence on the tendon's healing process is unclear.

**OBJECTIVE:**

To evaluate the influence of TENS on the healing of partial rupture of the Achilles tendon in rats.

**METHOD:**

Sixty Wistar rats were submitted to a partial rupture of the Achilles tendon by direct trauma and randomized into six groups (TENS or Sham stimulation) and the time of evaluation (7, 14, and 21 days post-injury). Burst TENS was applied for 30 minutes, 6 days, 100 Hz frequency, 2 Hz burst frequency, 200 µs pulse duration, and 300 ms pulse train duration. Microscopic analyses were performed to quantify the blood vessels and mast cells, birefringence to quantify collagen fiber alignment, and immunohistochemistry to quantify types I and III collagen fibers.

**RESULTS:**

A significant interaction was observed for collagen type I (p=0.020) where the TENS group presented lower percentage in 14 days after the lesion (p=0.33). The main group effect showed that the TENS group presented worse collagen fiber alignment (p=0.001) and lower percentage of collagen III (p=0.001) and the main time effect (p=0.001) showed decreased percentage of collagen III at 7 days (p=0.001) and 14 days (p=0.001) after lesion when compared to 21 days.

**CONCLUSIONS:**

Burst TENS inhibited collagen I and III production and impaired its alignment during healing of partial rupture of the Achilles tendon in rats.

## Introduction

Partial rupture of the Achilles tendon and tendinopathologies are frequent lesions and their incidence has grown due to the increase in sports practice by the general population^1^. These lesions are described in the literature as debilitating and difficult to repair because of the tendon's low metabolic rate^2^ and poor vascularization^3^, predisposing the patient to recurrence of the lesion.

Experimental studies have shown beneficial effects on vascularization, collagen fiber alignment, collagen type present in the tissue, and tensile force of the tendon after the application of electrophysical agents during the tendon healing process, such as low level laser therapy^4^, ultrasound^5^^,^ 6, electromagnetic fields^7^, and electrical stimulation^8^.

Among these resources, transcutaneous electrical nerve stimulation (TENS) can be highlighted as a simple, non-invasive, low-cost method that, in addition to modelling pain perception, has effects on local blood flow^9^^,^ 10, wound healing^11^, and reduction of the necrotic area of ischemic skin flaps^12^.

TENS can be used in four modalities: high-frequency (conventional), low-frequency (acupuncture), burst (with high frequency bursts, emitted at very low frequencies), and intense TENS^13^. However, according to Sluka and Walsh^9^, only the low-frequency and burst modalities are capable of reaching the A-delta and C nociceptive fibers, generating an increase in the local blood flow.

In addition to the vascular alterations, the healing effects probably occur because these nociceptive fibers, which signal pain to the central nervous system, are stimulated and carry a reflex to the peripheral nerve terminations, stimulating the release of neuropeptides^14^^,^ 15, such as Substance P (SP) and Calcitonin Gene Related Peptide (CGRP), with pro-inflammatory and healing effects^11^^,^ 15.

In order to stimulate these nociceptive fibers, some authors have demonstrated favorable effects on tendon healing after using TENS^16^^,^ 17. However, although it is a resource widely used in the treatment of acute and chronic pain, there is little reported in the literature regarding the histological alterations that this modality may cause during the healing process. We hypothesized that TENS can change the vascularization, mast cell content and collagen composition, and organization during the healing process. Thus, the objective of the present study was to analyze the influence of TENS on histological aspects of the tendon healing process.

## Method

This study was carried out with 60 male Wistar rats (*Rattus norvegicus: var.albinus, Rodentia, Mammalia*), 8-9 weeks of age, 260-320g body mass. The animals were kept individually in polypropylene cages with a 12-hour light-dark cycle and at constant 22 °C. They were given suitable ordinary feed as well as water ad libitum. All the experiments were approved by the Research Ethics Committee (protocol number 0591/11) of Universidade Federal de São Paulo (UNIFESP), São Paulo, Brazil, and the guidelines of the Association for Assessment and Accreditation of Laboratory Animal Care (AAALAC) were followed.

An equal number of rats were randomly assigned to six groups: SHAM-7, SHAM-14, SHAM-21, TENS-7, TENS-14, and TENS-21. For the randomization procedure, the rats were numbered on their tail with a pen for identification and then a randomization table was generated using the website www.randomization.com, which indicated to which group the animal should belong. A partial tendon rupture was induced by direct trauma in all animals. Shortly after, the three Sham groups received sham stimulation and the three TENS groups received TENS treatment, both for 6 consecutive days, beginning on the same day as the rupture. The Achilles tendons were removed and examined 7, 14 and 21 days after trauma-induced injury, and the animals were then euthanized.

### Lesion procedure

The animals were weighed and anesthetized with an intraperitoneal injection of ketamine hydrochloride (100 mg/kg) and xylazine hydrochloride (50 mg/Kg). The hair over and around the right Achilles tendon and dorsal region was shaved.

The animal's right paw was positioned on the injury device and a light traction was applied to the calcaneal region with the ankle in dorsiflexion until the dorsal surface of the paw touched the base of the device. Then, a weight of 186 g was released perpendicularly on the tendon of the animal from a height of 20 cm, corresponding to a potential energy of 364.9 mJ^6^^,^ 18.

Immediately after this procedure, the weight was removed, electrodes were positioned on the paw, and TENS or sham treatment was started. The animals were then returned to their cages and observed until the anesthesia wore off.

### TENS treatment

**Figure 1.**
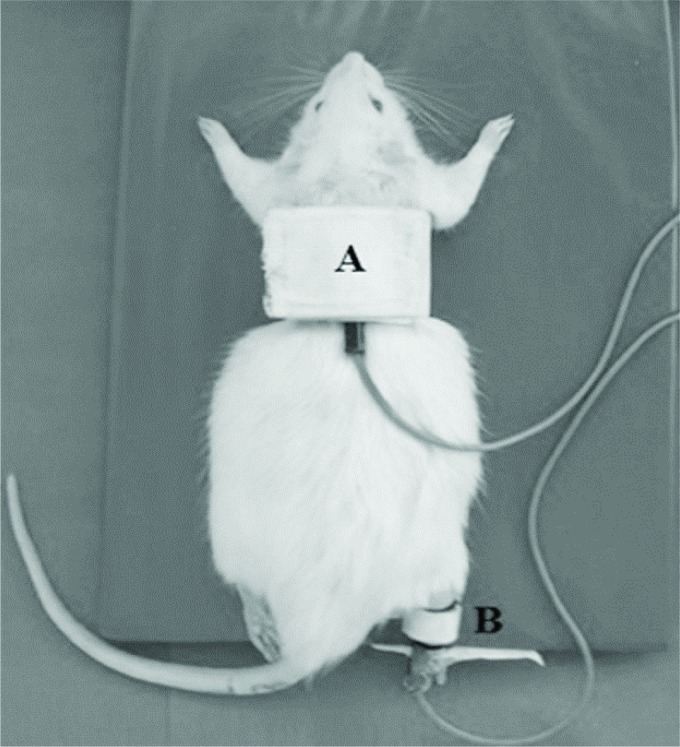
Positioning of the animal for the application or simulation of TENS. Electrodes placed (A) on the back of the animal, fixed by an elastic band, and (B) of the Achilles tendon, fixed by Velcro^®^

A TENS apparatus (Orion Tens^®^; Campinas, SP, Brazil) was used that transmitted a pulsed symmetrical rectangular biphasic current through two silicone rubber electrodes with a conducting gel (carboxyvinyl polymer) between the electrode and skin contact surfaces. Electrodes with an area size 5.5 cm x 3.2 cm were placed on the back of the animal starting at the upper edge of the scapula, and 3.2 cm x 1.8 cm, placed on the right hind paw on the Achilles tendon (Figure 1).

The treatment protocol began in the first minute after the lesion, and the animals placed in the TENS group were submitted to burst TENS at 100 Hz frequency, modulated at a frequency of 2 Hz, 200 µs pulse duration, and 300 ms pulse train duration for 30 minutes, with the intensity adjusted to 90% of the motor threshold of each animal (which was considered the first visible muscle contraction produced by TENS from its intensity increase). In the sham group, the electrodes were placed on the Achilles tendon and on the back of the animal for 30 min, but no current was transmitted (the equipment remained off).

In both groups, the rats were kept anesthetized with an intraperitoneal injection of ketamine hydrochloride (100 mg/Kg) and xylazine hydrochloride (50 mg/Kg) during the intervention, which was carried out for six consecutive days. The animals were euthanized on the 7^th^, 14^th^, and 21^st^ days post-lesion, according to their allocated subgroup.

### Tendon excision and sample preparation

The animals were anesthetized and the injured tendon of all the animals was surgically removed by dissection from the calcaneal insertion up to the muscle-tendon junction. Then, the animals were euthanized by anesthesia overdose.

The Achilles tendon was fixed to a cork surface using two pins; the first pin was placed on the base of the tendon (osteotendinous junction), and the second pin was placed on the gastrocnemius muscle. The sample was then fixed in 10% buffered formaldehyde for 40 minutes, after which each sample was wrapped in filter paper and placed inside a small box, which was immersed in 10% buffered formaldehyde for 24 h. Next, the tendons were immersed in 70% alcohol for 48 h, and then embedded in paraffin blocks, beginning the preparation of histological slides for vascularity evaluation with hematoxylin and eosin staining. Mast cells were stained with Toluidine blue and collagen evaluation with birefringence and immunohistochemistry (collagen I and III).

In order to perform the analysis, the histological slices were chosen randomly and the investigator did not know to which group each sample belonged (blind analysis).

### Assessment of blood vessels and mast cells

This measurement was taken from microphotographs of each slide using an image capture system (AxioVision^®^, Carl Zeiss, Germany) connected to an image analysis program (AxioVision^®^ 4.8REL) on the computer. The microphotographs were taken at 400x magnification (40x magnification lens and 10x eye lens - area of 0.622 mm^2^/field), after identifying the central region of the tendon, followed by a further two areas in sequential fields, one towards the median edge and the other towards the lateral edge, totaling three microphotographs of each slide. Using these captured images, the individual vessels and mast cells were counted, and the mean of the diameter of the vessels of the images of each animal was analyzed.

### Birefringence assessment

Polarized light microscopy was used to measure the anisotropic properties (birefringence) of the collagen fibers. Optical delay (OR in nm) measurements were taken with polarized light microscopy in a Leica^®^ microscope, with a Pol 10x/0.22 lens, 0.9 condenser, 1/4 lambda Sénarmont compensator, 546 nm monochromatic light, obtained by means of a Leica^®^ interference filter.

In order to take the measurement, the long axis of the tendon was kept at approximately 45° in relation to the microscope polarizers. OR measurements were taken at three different points in the central areas of the tendons and the average was used to calculate. The resulting measurements in degrees were converted to nm by multiplying the degrees by 3.03.

### Collagen I and III assessment

The expression of collagen I and III, observed in the immunohistochemical reaction with the polyclonal antibodies for the collagen types I and III (Affinity Purified Anti-Collagen Type I and Type III, Rabbit Anti-Human, Rockland Inc., Gilbertsville, PA, USA), was analyzed by quantifying the area corresponding to each type of collagen. For this assessment, three images were obtained representative of the central region of the tendon magnified 400x (40x lens), using a CoolSNAP-Pro cf camera (Media Cybernetics Inc.) attached to a Nikon Eclipse-E800 microscope. After obtaining the images, the marked area was identified by a colorimetric recognition tool, using the Image-ProPlus computerized image system, version 4.5 (Media Cybernetics Inc). The marked areas were then added by slide and the percentage of collagen was calculated compared to the total area of the tendon analyzed.

### Statistical analysis

The Shapiro-Wilk test was used to evaluate the data distribution, and all the variables showed p<0.05. A 2-factor ANOVA was conducted for all variables with time (7, 14 and 21 days) as within factor and group (TENS and sham) as between factor. The Tukey test was used for post-hoc analysis when necessary. If no time x group interaction was observed, the main effects of time and groups were analyzed. The statistical analysis of the results was carried out in the R program, version 2.15.3. The level of significance was set at p<0.05.

## Results

**Figure 2.**
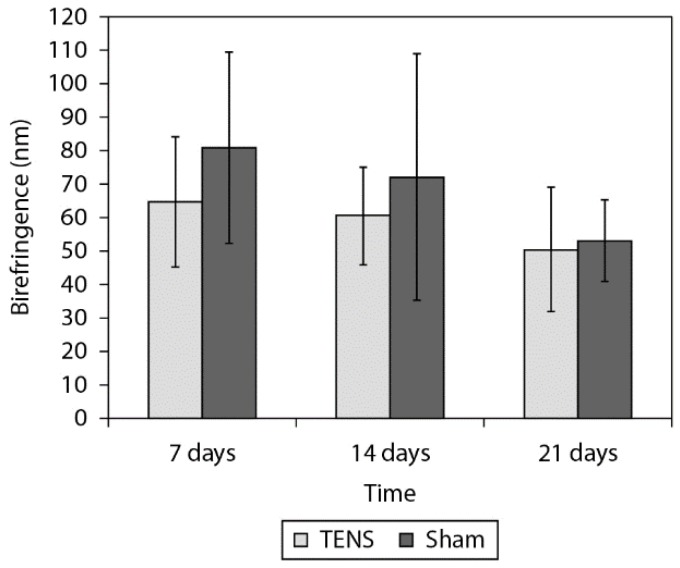
Comparison of the birefringence (nm) analysis on the tendon tissue between the TENS and Simulation groups. Data expressed in mean and standard deviation.

**Figure 3.**
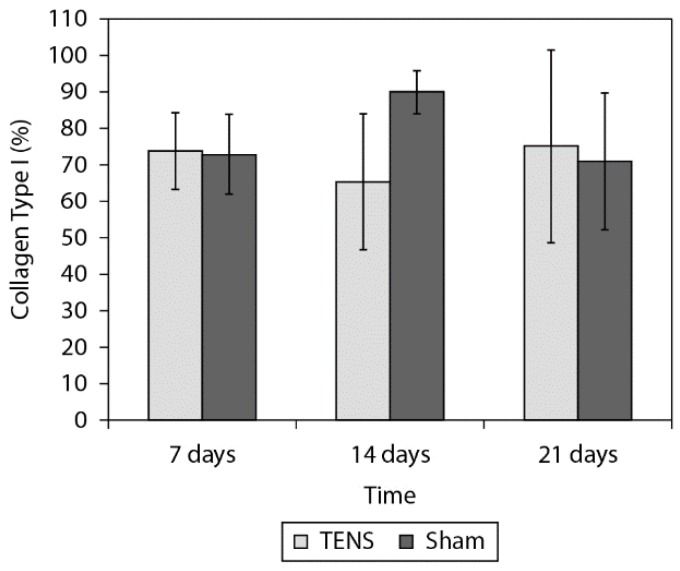
Comparison of the quantity of collagen types I (%) in the tendon tissue between the TENS and Simulation groups. Data expressed in mean and standard deviation.

**Figure 4.**
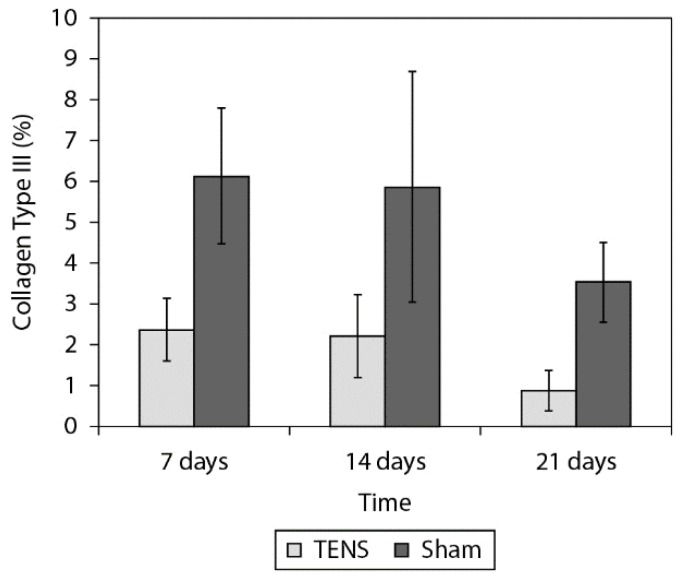
Comparison of the quantity of collagen types III (%) in the tendon tissue between the TENS and Simulation groups. Data expressed in mean and standard deviation.

Figures 2, 3and 4 show the birefringence, quantity of collagen type I and type III, respectively, for both groups at all times. Table 1 shows the ANOVA results. The 2-factor interaction was not significant for any variables (Table 1) except for collagen type I (p=0.020). After the Tukey Post hoc test was possible observe that the TENS group presented lower percentage of collagen type I 14 days after the lesion (p=0.033, Figure 3). The main effect of group showed that the TENS group presented worse collagen fiber alignment (p=0.001) and lower percentage of collagen III (p=0.001) than the Sham group. The main effect of time (p=0.001) showed decreased percentage of collagen III at 7 days (p=0.001) and 14 days (p=0.001) after lesion when compared to 21 days.

**Table 1.** Measures of variables related to group, assessment, and results of Analysis of Variance (ANOVA).

Variables	Group	Time	Average	Standard deviation	Group		Time		Interaction	
					p value	F value	p value	F value	p value	F value
Vessels	SHAM	7	10.90	5.53	0.612	(1;54)=0.26	0.885	(2;54)=0.12	0.189	(2;54)=1.72
		14	9.90	3.11						
		21	9.10	5.00						
	TENS	7	8.90	2.42						
		14	11.10	2.60						
		21	11.40	3.03						
Diameter vessels	SHAM	7	10.09	2.51	0.600	(1;53)=0.27	0.382	(2;53)=0.95	0.741	(2;53)=0.30
		14	10.07	2.83						
		21	9.12	2.07						
	TENS	7	8.89	2.07						
		14	10.43	4.72						
		21	8.66	3.60						
Mast cells	SHAM	7	4.20	3.33	0.533	(1;54)=0.39	0.213	(2;54)=1.51	0.883	(2;54)=0.12
		14	3.20	2.82						
		21	2.20	2.30						
	TENS	7	4.40	2.41						
		14	3.40	3.89						
		21	3.20	2.49						
Birefringence	SHAM	7	80.75	28.57	0.001	(1;52)=13.03	0.196	(2;52)=1.68	0.377	(2;52)=0.99
		14	64.54	19.44						
		21	87.57	60.14						
	TENS	7	60.45	14.64						
		14	53.18	12.40						
		21	50.30	18.51						
Collagen Type I	SHAM	7	72.94	10.85	0.184	(1;36)=1.83	0.589	(2;36)=0.54	0.020	(2;36)=4.36
		14	90.00	5.97						
		21	71.18	18.74						
	TENS	7	73.94	10.59						
		14	65.54	18.72						
		21	75.18	26.27						
Collagen Type III	SHAM	7	6.11	1.65	0.001	(1;48)=89.23	0.001	(2;48)=18.52	0.101	(2;48)=2.40
		14	5.85	2.82						
		21	3.53	0.97						
	TENS	7	2.36	0.77						
		14	2.19	1.01						
		21	0.86	0.49						

## Discussion

TENS became popular in the 1960s after a study by Melzack and Wall^19^ that elucidated its main action mechanism: the gate theory. It is now known that its action mechanisms are more complex and include anatomic pathways, some types of neurons and neurotransmitters, and their receptors^9^, leading to effects that go beyond hypoalgesia, such as increase in cutaneous temperature and local blood flow^10^, and even healing effects^12^^,^ 20 ^,^ 21.

Some studies have reported positive effects of TENS on tendon healing using the high frequency conventional modality^20^^,^ 21. However, Sluka and Walsh^9^ stated that only acupuncture at low frequency and burst modalities are capable of reaching the A-delta and C nociceptive fibers, generating vascular and healing effects resulting from neurogenic inflammation. According to Sjölund^22^, the ideal modality to stimulate the nociceptive fibers mentioned above is the burst mode, which justifies its use in the present study.

In spite of this premise, in the present study no relevant difference was detected among the groups regarding the quantity and diameter of the vessels, in all the times. These findings corroborate results by Burssens et al.^16^, who also did not report effects regarding vascularization from burst mode TENS application in the human tendon. However, it is important to point out that, both in the study by these authors and in the present study, the intensity of the TENS used was below the motor threshold, and according to Machado et al.^23^, it is only possible to reach an important increase in local blood flow when the intensity applied is above 25% of the motor threshold. Although Machado et al.^23^ based their studies on cutaneous injuries, intensity adjustment is a factor that can justify the absence of effects regarding vascularization.

Mast cell degranulation is also an important event in the healing process, because it releases mediator cells that stimulate the extracellular matrix synthesis^24^. Thus, stimulating mast cell proliferation with TENS could be advantageous for the tendon healing process, but no difference was detected among the groups studied. A study observed a higher quantity of mast cells in tendon tissue after applying LLLT after the same lesion procedure as utilized in the present study^18^, but no other study was found in the literature researched that assessed the quantity of mast cells from using TENS on the tendon healing.

The absence of effects from TENS regarding the mast cells and the absence of vascular effects allows the inference that TENS (with the parameters adopted in the present study) was not able to reach the nociceptive fibers and stimulate SP and CGRP release, because these neuropeptides have not only vasoactive and angiogenic effects but also the ability to stimulate mast cell proliferation and degranulation^25^.

Another aspect of fundamental importance in studies on the tendon healing process is collagen, because this structure is the main component of the extracellular matrix and confers on the tendon the ability to support and transmit great forces between the muscle and bone^26^. One of the factors responsible for this resistance of the tissue against the forces of tension is the organization of the collagen fibers that, under normal conditions, are placed parallel along the longitudinal axis of the tendon^27^.

In the present study, the TENS groups presented less alignment of the collagen fibers compared to the Sham groups in all the times. These findings did not corroborate results by Burssens et al.^17^, who verified in patients with ruptured Achilles tendon a better alignment of the collagen fibers in the six-week period after applying burst TENS. However, in the study by Burssens et al.^17^, the patient's ankle remained immobilized throughout the study period, while the present study allowed free movement of the animal's pelvic members. In this case, the absence of a controlled charge applied early to the lesion and member may have damaged the quality of the tissue.

In addition to the collagen fiber alignment, the type of collagen present in the tissue also interferes with the tendon's capacity to resist forces between the muscle and bone. The main collagen fibers present in the tendon are type I, with greater caliber and contribution to tissue resistance, and type III, with smaller caliber, less organized fibrils, and less resistant tension forces. In injured tendons, the tenocytes tend to produce a greater quantity of collagen type III, causing a reduction in the proportion of these fibers that may result in a tendon less resistant to stress and with greater risk of new ruptures^28^^,^ 29.

In the present study, the TENS groups, compared to the Sham groups, presented a smaller quantity of collagen type I in the 14-day post-lesion period (p=0.020) and a smaller quantity of collagen type III in the periods of 7 days (p=0.001), 14 days (p=0.001), and 21 days (p=0.001) post-lesion. In the long term, a smaller quantity of collagen type III fibers obtained from applying TENS may be considered a beneficial effect, if it was accompanied by the stimulation of collagen type I fiber production because it would give the tissue greater tension-resisting capacity.

On the other hand, reduction in the quantity of collagen type III fibers, without increase in the quantity of collagen type I fibers, led to the inference that the tissue presented a smaller total quantity of collagen that could damage the tension force of this tissue, especially in the 14 day post-lesion period when the TENS group presented a smaller quantity of collagen fibers of both types.

It is known that one of the factors that can contribute to the occurrence of physiological effects is the density of the current applied to the tissue, following the theory based on the law by Arndt-Schultz that states that excess energy may lead to deleterious effects to the tissue^30^. This fact may have happened in the present study regarding the stimulation intensity, generating inhibition in collagen I and III production and worsening the fiber alignment.

No other study was found in the literature that used TENS after tendon rupture and evaluated the quantity of collagen types I and III fibers in a specific way. However, Burssens et al.^16^ and Araújo et al.^20^ reported an increase in the quantity of tenocytes, which synthesize the collagen fibers, after applying TENS. Sharifi et al.^21^ observed a greater quantity of hydroxyproline in tendon tissue after using TENS, and as this amino acid is present in large quantities in collagen, the authors concluded that TENS led to increased collagen production in the tendon. Burssens et al.^17^observed a greater quantity of newly formed collagen and an earlier maturation of these fibers in the tendon after applying TENS. The present study did not corroborate these findings; however, the stimulation intensity varied between the studies cited, and although the majority remained at a sensitive threshold intensity^16^^,^ 17 ^,^ 20, none of these authors described the area of the electrodes used. Therefore, it is impossible to calculate the density of the current used (density = intensity / area) to make some concrete comparison with the present study.

There is another possible justification considering the main effect of TENS, analgesia. Electrical stimulation might have reduced the pain caused by the tendon lesion permitting the animal to move the wounded limb more than the other group. This fact may have damaged the healing process of this tissue. In order to prevent the influence of pain on the animal's movement, it is recommended that the wounded limb be immobilized in future studies on electrophysical resources in tendon healing.

The present study showed some limitations that are worth discussing. A control group (injury without intervention) was not included, and it is important to note that even a treatment simulation (Sham group) may have some effect on the variables studied. However, we believe that some comparisons between groups could be made because all procedures on TENS groups were conducted in the Sham group. We followed strict rules about the same stress factors during handling, the same anesthetic dosage in all treatment procedures, the same conductive gel, and the same type and size of electrodes. The electrical stimulation was the only difference between the groups, and if the results show relevant differences between them, it is probably due to TENS. We believe that these groups were sufficient to demonstrate the TENS effects on tendon healing, and we followed the principles of ethics in animal testing using the smallest possible number of animals.

Another point that must be clarified is that although no study was found on the influence of ketamine hydrochloride and xylazine hydrochloride on electrical stimulation, the animals were kept anesthetized with these drugs during the treatments. We believe that these drugs do not interfere in TENS effects and this temporary mechanical restriction did not influence the results; however, even if this mechanical restriction did influence the tendon healing, both groups were submitted to the same procedures except for the TENS stimulation.

It is important to point out that this is an experimental study carried out on rats that elucidated some clinically relevant questions and, although it clarifies some physical and pathological aspects in the partial rupture of the tendon, the findings of the present study cannot be applied to humans, because there are differences between the tendons of these species, especially regarding the healing stage. Thus, clinical studies are needed but they should be carried out only with evidence that ensures TENS as a modality that favors tendon repair.

In conclusion, burst TENS had no effect on tissue vascularization and mast cell quantity, but it did influence the healing process of the partial rupture of the Achilles tendon in rats, with damage to the collagen fiber alignment. It also reduced the quantity of collagen type III and the quantity of collagen type I in the 14-day post-lesion.
